# Big Tongue

**Published:** 1897-07

**Authors:** J. Frank Thornton

**Affiliations:** Plum, Texas


### Big Tongue.

PLUM, TEXAS, June 7, 1897.

*Editors Texas Medical Journal:*

I write to ask if you ever saw or heard of a case like these I have on hand. You may give it a name. A child, a girl 3½ years old, commenced to talk at the age of 22 months, and has ceased to utter a word at all, owing to the thickness and breadth of the tongue, and it is growing larger every day. The tongue in this white girl is as large as that of a big black negro man. The mother informs me that when the child was born the tongue looked then to be abnormally large, but no attention was paid to it until the child commenced to stop talking by degrees; it never articulated very well, but has ceased to speak altogether. There is another child younger than this one who seems to be afflicted in the same way. You may publish if you think best.

Hoping to hear from you soon, I am fraternally yours,

J. FRANK THORNTON, M. D.

